# Utility of droplet digital polymerase chain reaction for *TERT* and *BRAF* mutational profiling of thyroid nodules

**DOI:** 10.1186/s12885-021-08810-8

**Published:** 2021-10-26

**Authors:** Brandon R. Rosvall, Morris Kostiuk, Jordana Williams, Ashlee Matkin, Jeffrey Harris, Hadi Seikaly, Daniel A. O’Connell, Vincent L. Biron

**Affiliations:** 1 Division of Otolaryngology - Head and Neck Surgery, Department of Surgery, University of Alberta, 1E4 Walter Mackenzie Center, 8440 112 St, Edmonton, Alberta T6G 2B7 Canada; 2 Division of Plastic and Reconstructive Surgery, Department of Surgery, University of Alberta, Edmonton, Alberta Canada

**Keywords:** Thyroid, Oncology, Cancer, Cytology, PCR, ddPCR, Molecular, Mutation, BRAF, TERT

## Abstract

**Background:**

Mutations involving BRAF and TERT are important predictors of disease severity in thyroid cancer, but molecular testing is limited by cost and lack of adequate tissue sample. This study aimed to assess the utility of BRAFV600E and TERT testing using droplet digital PCR (ddPCR) as a diagnostic and prognostic tool for thyroid fine needle aspirate biopsy (FNAB).

**Methods:**

Patients with thyroid nodules were prospectively enrolled from March 2015 to September 2018. Pre-operative FNAB was collected for standard cytology and molecular testing. BRAFV600E and TERT levels were analyzed by ddPCR. Cytology (Bethesda system) and ddPCR results were correlated to surgical pathology.

**Results:**

A total of 222 patients were enrolled, of which 124 received thyroid surgery. Pre-operative cytology alone with Bethesda ≥5 was 100% specific and 70% sensitive for malignancy on final surgical pathology. BRAFV600E positivity or TERT overexpression was 100% specific and 60.0% sensitive. Combining cytology (Bethesda ≥5) with BRAFV600E and TERT testing increased the sensitivity of a malignant diagnosis to 80.0%. High TERT levels and/or BRAFV600E was associated with aggressive or advanced stage pathology.

**Conclusions:**

Combining cytology with ddPCR analysis of BRAFV600E and TERT can improve the diagnostic accuracy of thyroid FNAB, and help predict aggressive pathology.

**Supplementary Information:**

The online version contains supplementary material available at 10.1186/s12885-021-08810-8.

## Background

In recent years, the incidence of thyroid cancer has markedly increased worldwide [1, 2]. While the prognosis of thyroid cancer is generally favorable, there is a five to 20% rate of local recurrence, and 10 to 15% rate of distant metastases [2–4]. Whereby repeat operations and more extensive surgery are associated with increased morbidity, improved prognostication of thyroid nodules may result in improved patient outcomes [5].

The 2015 American Thyroid Association (ATA) guidelines recommend workup of thyroid nodules using ultrasound followed by fine needle aspirate biopsy (FNAB) if warranted Bethesda cytopathologic categories are used to risk stratify thyroid nodules and guide management; however, this system is limited in its diagnostic accuracy [6]. ATA guidelines suggest that molecular testing may supplement malignancy risk assessment in indeterminate thyroid nodules categorized Bethesda III-V.

Several gene alterations have been identified as important biomarkers of thyroid cancer with varying sensitivity and specificity [7]. In well-differentiated thyroid cancer, telomerase reverse transcriptase (TERT) promoter and B-type raf proto-oncogene V600E (BRAFV600E) mutations have demonstrated particular utility in predicting disease and high risk clinicopathology. The BRAFV600E mutation has been shown to exert oncogenic potential through a mitogen-activated protein kinase dependent process in which it increases susceptibility to a transforming growth factor β mediated epithelial–mesenchymal transition [8]. The BRAFV600E mutation is the most prevalent mutation associated with thyroid cancer, found in nearly half of papillary thyroid cancers [9, 10]. It has a high specificity and positive predictive value for thyroid cancer, is related to aggressive thyroid cancer subtypes and predicts a worse overall prognosis including increased mortality [7, 9–13]. TERT is responsible for the maintenance of telomere length at the end of chromosomes, through which it influences cellular proliferation and immortality [14]. TERT expression can be upregulated through several mechanisms including promoter mutations, TERT gene copy number alterations, increased promoter methylation and histone modifications [14–18]. These TERT-related genetic aberrations have been implicated in thyroid cancer with particular prevalence in aggressive subtypes [15, 16]. They have a high specificity and positive predictive value for thyroid cancer [19] and have been associated with poorer patient outcomes, including a greater risk of death [14, 15, 20].

BRAF and TERT mutations are both predictors of high risk pathology in the ATA guidelines. These mutations can be tested for in a number of commercially available molecular tests, however these are often costly and require large volumes of RNA which can be difficult to obtain with FNAB [21, 22]. Droplet digital PCR (ddPCR) is an ultrasensitive method of detecting gene targets, with advantages over other molecular techniques in specimen containing low amounts of nucleic acid [23–29]. Pre-operative FNAB molecular testing of thyroid nodules by ddPCR has recently been validated for BRAFV600E and RAS mutations but did not include TERT [30]. In this study, TERT overexpression was measured *in lieu* of TERT promoter mutations to capture a broader range of TERT upregulatory mechanisms.

There is a paucity of research assessing the utility of BRAFV600E and TERT for profiling thyroid nodules using ddPCR techniques. This study aimed to assess the utility of BRAFV600E and TERT ddPCR testing as a diagnostic adjunct for thyroid FNAB.

## Methods

### Study enrolment

Patients presenting to the University of Alberta Head and Neck Surgery Clinic were prospectively recruited and consented for enrolment in the study from March 2015 to September 2018. Patients were eligible for study enrolment if they had a thyroid nodule meeting indication for FNAB as per the 2015 ATA guidelines [6]. The University of Alberta Health Research Ethics Board provided ethics approval for the study protocols (Pro00062302 and Pro00016426). The study conformed with The Code of Ethics of the World Medical Association (Declaration of Helsinki) and was undertaken with the understanding and written consent of each subject.

### Fine needle aspirate biopsy specimen

An ultrasound-guided FNAB was performed as standard of care for cytology, with an additional sample taken for ddPCR analysis. Samples were immediately transferred to a 1.5 mL tube containing 200 μl RNAlaterTM (Thermofisher AM7021) and kept at room temperature < 24 h and at 4 °C for < 7 days until processed for RNA extraction. Determination of BRAFV600E and TERT levels by ddPCR was performed by MK, who was blinded to clinical and pathologic characteristics associated with FNAB samples. Surgical treatment decisions were made according to the 2015 ATA guidelines and were not influenced by ddPCR mutation results [6].

### Nucleic acid preparation

RNA was extracted using the RNeasy PlusMini Kit (Qiagen Cat No./ID: 79656). 550 μl of Buffer RLT, 40 mM DTT was added directly to the tube containing the FNAB and the tube was vortexed extensively. The sample was loaded onto a QIAshredder (Qiagen Cat No./ID: 79656) and centrifuged at 8000 x g for 30 s at room temperature. The resulting flow through was loaded onto a gDNA Eliminator mini Spin Column and centrifuged 30 s at 8000 x g. An equal volume of 70% ethanol was added to the flow through, mixed by pipetting, and the mixture was transferred to a RNeasy Mini spin column and centrifuged for 15 s at 8000 x g. Following RNA binding, the Mini column was washed as per manufacturer’s instructions and the RNA was eluted with 50 μl RNase free H_2_O. RNA concentration was quantified using the Qubit RNA HS assay kit on a Qubit 2.0 fluorometer as per manufacturer’s instructions. The RNA was either stored at -80o C or immediately used to carry out cDNA synthesis.

RNA (5–500 ng) was used to synthesize cDNA using the iScriptTM Reverse Transcription Supermix for RT-qPCR (BIO-RAD) as per the manufacturer’s protocol. Following the reaction, the cDNA was diluted with nuclease free H_2_O to a final concentration of 1 ng/μl (if initial RNA concentration was high enough) or, in some cases, 2 ng/ul. Newly synthesized cDNA was either stored at -20 °C or used directly for ddPCR.

### Droplet digital polymerase chain reaction and analysis

Reactions were set up following the manufacturer’s protocols using 12 μl/reaction of 2× ddPCR Supermix for Probes (No dUTP), 1.2 ul/reaction of 20× mutant primers/probe (FAM BIO-RAD), 1.2 μl/reaction 20× wildtype primers/probe (HEX, BIO-RAD), 2.4 ul cDNA (at up to 2 ng/ul) and 7.2 μl H2O. ddPCR was carried out using the ddPCRTM Supermix for Probes (No dUTP) (BIO-RAD), the QX200TM Droplet Generator (catalog #186–4002 BIO-RAD), the QX200 Droplet Reader (catalog #186–4003 BIO-RAD) the C1000 TouchTM Thermal Cycler (catalog #185–1197 BIO-RAD) and the PX1TM PCR Plate Sealer (catalog #181-40well plate, mixed using a Mixmate Vortex Shaker (Eppendorf) and 20 ul of the reaction mixture was transferred to DG8TM Cartridge for QX200/QX100 Droplet Generator (catalog #186–4008 BIO-RAD) followed by 70 μl of Droplet Generation Oil for Probes (catalog #186–3005 BIO-RAD) into the oil wells, according to the QX200 Droplet Generator Instruction Manual (#10031907 BIO-RAD). Following droplet generation, 40 ul of the reaction was transferred to wells of a 96 well plate and the reactions were carried out in the thermocycler using the following parameters: Step 1) 95^o^ C for10min, Step2) 94^o^ C for 30s and 60^o^ C for 1 min (Step 2 repeat 39 times for a total of 40), Step 3) 98^o^ C for 10 min and Step 4) 4^o^ C infinite hold. All steps had a ramp rate of 3^o^ C/second. Following thermocycling the reactions were read in the QX200 Droplet Reader and the RNA targets were quantified using the QuantasoftTM Software (BIO-RAD).

BIO-RAD proprietary ddPCR Primers and probes used were as follows: Unique Assay ID dHsaCP2000037 PrimePCR ddPCR Mutation Assay BRAF p.V600R Human (FAM), Unique Assay ID dHsaCP2000028 PrimePCR ddPCR Mutation Assay BRAF WT for p.V600E Human (HEX), Unique Assay ID dHsaCPE5048434 PrimePCR ddPCR Assay TERT for Human (FAM) and Unique Assay ID dHsaCPE5050049 PrimePCR ddPCR Assay EEF2 for Human (HEX).

**Fig. 1:**
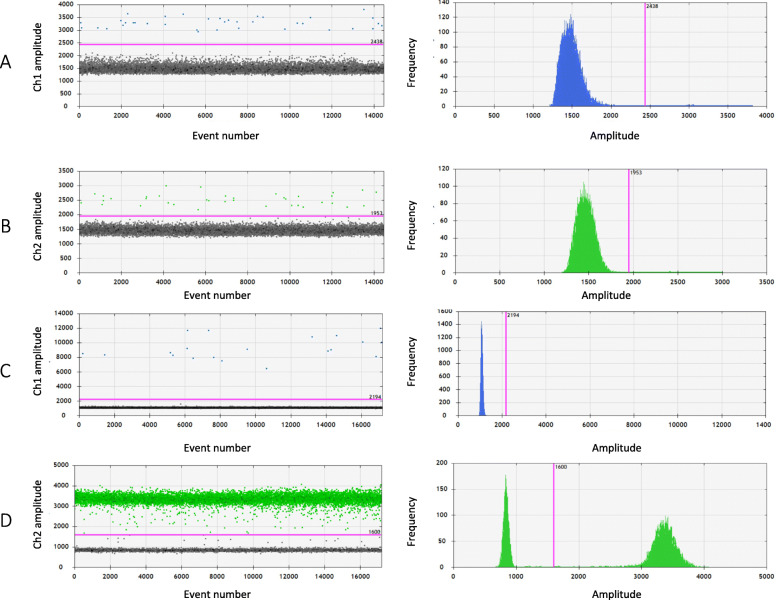
Droplet digital PCR analysis of BRAFV600E and TERT. Detection of BRAF mutation shown in a patient with A) BRAF V600E (FAM) and B) corresponding wildtype BRAF copy (HEX). Droplets positive for BRAF V600E are shown in blue with an amplitude shift upwards while the background negative droplets are grey. Droplets positive for wildtype BRAF are shown in green with the negative droplets in grey. C) TERT expression (FAM) compared to D) gene expression control EEF2 (HEX). Droplets positive for TERT are shown in blue with an amplitude shift upwards while the background negative droplets are grey. Droplets positive for EEF2 are shown in green with the negative droplets in grey

Determination of mutant versus wild type BRAF samples was based on the presence or absence of mutant droplets in the expected regions in two-dimensional data output plots determined using Quantasoft (Fig. 1). TERT abnormality was determined by thresholding expression of TERT at 10% of the lowest value in the range of values in normal and cancerous specimen. This cutoff value was chosen because 1) a small number of normal samples had detectable ddPCR TERT expression < 10% of max values 2) this level of expression measured by ddPCR is the limit of detection seen by qRT-PCR in our previous studies. EEF2 was used as a gene expression control, correcting for differences in overall gene expression.

### Statistics

Statistical calculations were completed using SPSS version 25 (IBM, Chicago, IL) and MedCalc version 19 (MedCalc Software, Ostend, Belgium) where appropriate. Bayesian statistics were used to calculate means, Pearson correlation and Loglinear regression. Non-parametric comparisons were made between groups of thyroid cancer specimen. The performance of standard cytology (Bethesda classification) and ddPCR to correlate with thyroid cancer on surgical pathology was estimated using Bayes theorem. Where appropriate, 95% confidence intervals were calculated using Clopper-Pearson for sensitivity and specificity, the Log method for positive likelihood ratios (PLR), negative likelihood ratios (NLR), positive predictive value (PPV) and negative predictive value (NPV) [31].

## Results

A total of 124 patients underwent thyroid surgery, of whom 86.3% (107) were female, 51.6% (64) were 55 years of age or older, and 23.4% (29) had a nodule 4.0 cm or larger (Table 1).

**Table 1:** Characteristics of patients who received thyroid surgery in this study

Variable	All (%), ***N*** = 124	Bethesda Categories (%)					
		I	II	III	IV	V	VI
		(non-dx)	(benign)	(AUS/FLUS)	(FN/SFN)	(SFM)	(malignant)
		***N*** = 28	***N*** = 51	***N*** = 9	***N*** = 8	***N*** = 5	***N*** = 23
**Age**							
*Mean (SD)*	53.6 (14.6)	54.9 (12.0)	52.4 (15.8)	52.6 (15.1)	65.8 (13.3)	49.0 (4.6)	52.1 (15.6)
*< 55*	60 (48.4)	10 (35.7)	24 (46.1)	5 (55.5)	2 (25.0)	5 (100)	14 (60.9)
*>* *55*	64 (51.6)	18 (64.3)	27 (53.9)	4 (44.5)	6 (75.0)	0	9 (39.1)
**Sex (female)**	107 (86.3)	27 (96.4)	47 (90.3)	8 (88.9)	4 (50.0)	3 (60.0)	18 (78.2)
**Nodule size**							
*1.0–3.9 cm*	95 (76.6)	28 (100)	38 (74.5)	0	5 (62.5)	3 (60.0)	20 (86.9)
*≥ 4.0 cm*	29 (23.4)	0	13 (25.5)	9 (100)	3 (37.5)	2 (40.0)	3 (13.1)
**Operation**							
*Lobectomy*	84 (73.9)	22	44	6	8	0	4
*Total +/− Level 6 ND*	35 (25.0)	6	7	2	0	5	15
*Total + LND*	5 (4.0)	0	0	1	0	0	4
**Surgical pathology**							
*Benign*	84 (67.7)	24	46	7	7	0	0
*PTC*	31 (25.0)	3	4	1	1	4	18
*FTC*	3 (2.4)	1	1	1	0	0	0
*ATC*	6 (4.8)	0	0	0	0	1	5

*ATC* anaplastic thyroid cancer; *AUS* atypia of unknown significance; *dx* diagnostic; *FLUS* follicular lesion of unknown significance; *FN* follicular neoplasm; *FTC* follicular thyroid cancer; *LND* lateral neck dissection; *ND* neck dissection; *PTC* papillary thyroid cancer; *SD* standard deviation; *SFN* suspicious for follicular neoplasm

There were 88 patients who underwent lobectomy, 31 who underwent total thyroidectomy with or without level VI neck dissection, and 5 who underwent total thyroidectomy with lateral neck dissection. FNAB results from standard of care cytology yielded the following distribution in Bethesda classification: 22.6% (28) nondiagnostic, 41.1% (51) benign, 7.3% (9) atypia/follicular lesion of undetermined significance, 6.5% (8) follicular neoplasm or suspicious for follicular neoplasm, 4% (5) suspicious for malignancy, 18.5% (23) malignant (Table 2). FNAB ddPCR demonstrated BRAFV600E positivity in 16.1% (20) of nodules, TERT overexpression in 8.1% (10), and combined BRAFV600E positivity and TERT overexpression in 4.8% (6). On final surgical pathology, 67.7% (84) of nodules were benign and 32.3% (40) were malignant.

**Table 2:** Distribution of pre-operative fine needle aspirate cytology and ddPCR results according to final surgical pathology

Fine Needle Aspirate	Surgical Pathology		
	Benign (%), ***N*** = 84	Malignant (%), ***N*** = 40	Total (%), ***N*** = 124
**Cytology (Bethesda)**			
*I - Non-diagnostic*	24 (28.6)	4 (10)	28 (22.6)
*II - Benign*	46 (54.8)	5 (12.5)	51 (41.1)
*III - AUS/FLUS*	7 (8.3)	2 (5)	9 (7.3)
*IV - FN/SFN*	7 (8.3)	1 (2.5)	8 (6.5)
*V - SFM*	0	5 (12.5)	5 (4.0)
*VI - Malignant*	0	23 (57.5)	23 (18.5)
**ddPCR Result**			
*BRAFV600E*	0	20 (50)	20 (16.1)
*TERT overexpression*	0	10 (25)	10 (8.1)
*BRAFV600E + TERT*	0	6 (15)	6 (4.8)

*AUS* atypia of unknown significance; *FLUS* follicular lesion of unknown significance; *FN* follicular neoplasm; *SFN* suspicious for follicular neoplasm

Pre-operative cytology alone with Bethesda IV-VI was 91.7% specific and 72.5% sensitive for malignancy on final surgical pathology (Table 3). FNAB ddPCR results showing isolated BRAFV600E positivity and TERT overexpression were 50.0 and 25.0% sensitive, respectively, and each were 100.0% specific. The combination of BRAFV600E positivity, TERT overexpression, or both was 60.0% sensitive and 100% specific. Relative to pre-operative cytology alone, Bethesda IV-VI cytology, BRAFV600E positivity, or TERT overexpression increased the sensitivity of a malignant diagnosis to 80.0% while maintaining 91.7% specificity. Pre-operative Bethesda V-VI cytology, BRAFV600E positivity, or TERT overexpression maintained a sensitivity of a malignant diagnosis of 80.0% while increasing specificity to 100.0%.

**Table 3:** Comparative diagnostic performance of pre-operative standard cytology and ddPCR testing

Measure						**BRAFV600E + TERT +	**BRAFV600E + TERT +
	Bethesda	Bethesda	BRAFV600E	TERT	BRAFV600E	Bethesda	Bethesda
	IV-VI	V-VI			+TERT	IV-VI	V-VI
Sensitivity	72.5	70	50.0	25.0	60.0	80	80
Specificity	91.7	100	100	100	100	91.7	100
PPV*	80.6	100	100	100	100	82.1	100
NPV*	87.5	87.5	74.4	73.7	84.0	90.6	91.3
PLR	8.7	–	–	–	–	9.6	–
NLR	0.3	0.3	0.5	0.8	0.4	0.2	0.2

*NLR* negative likelihood ratio; *NPV* negative predictive value; *PLR* positive likelihood ratio; *PPV* positive predictive value. *Because the sample sizes in disease positive and disease negative groups may not reflect the true population prevalence of the disease, PPV and NPV may be inaccurate. **Combined BRAF and TERT classifies test as positive if BRAFV600E and/or TERT and/or Bethesda IV-VI or V-VI is present

A total of 40 thyroid cancers were included, of which 33 were papillary thyroid cancer, 3 were follicular thyroid cancer, and 4 were anaplastic thyroid cancer. There were 22 T1, 4 T2, 8 T3, and 6 T4 thyroid cancers (Table 4) [32]. BRAFV600E positivity was found in 50% (20) of thyroid cancers, TERT overexpressions was found in 25% (10) of thyroid cancers and dual positivity for BRAFV600E and TERT was found in 15% (6) of thyroid cancers (Table 2). No BRAFV600E positivity or TERT overexpression was identified in FNAB with associated benign surgical pathology.

**Table 4:** Pathologic features of 40 thyroid cancers associated with BRAFV600E and TERT overexpression

Pathology (*N* = 40)	Negative BRAF/TERT *N* = 16	BRAFV600E *N* = 20	TERT *N* = 10	BRAFV600E + TERT *N* = 6
**Tumor type**				
*PTC*	13	18	6	4
*FTC*	3	0	0	0
*ATC*	0	2	4	2
**Stage**				
*T-stage*				
*T1a*	8	4	1	0
*T1b*	3	5	3	2
*T2*	1	3	0	0
*T3*	3	5	2	2
*T4a*	1	1	0	0
*T4b*		2	4	2
*N-stage*				
*N0*	13	1	1	0
*N1a*	3	10	3	3
*N1b*	0	9	6	3
**LVI or PNI present (*n*** **= 14)**	2	10	3	1
**Extrathyroidal extension (*n*** **= 13)**	3	8	6	4
**Multifocal (*n*** **= 14)**	3	11	4	4

*ATC* anaplastic thyroid cancer; *FTC* follicular thyroid cancer; *LVI* lymphovascular invasion; *PTC* papillary thyroid cancer; *PNI* perineural invasion

Elevated TERT levels or dual positivity for TERT and BRAFV600E was associated with aggressive or advanced stage pathology (Table 5). TERT was positive in all four cases of anaplastic thyroid cancer. All TERT positive cases were associated with aggressive features and 40% were anaplastic. Of TERT positive cases, 30% (3) had lymphovascular or perineural invasion, 60% (6) had extrathyroidal extension, and 40% (4) had multifocal disease. Of cases with dual positivity for TERT and BRAFV600E, 16.7% (1) had lymphovascular or perineural invasion, 66.7% (4) had extrathyroidal extension, and 66.7% (4) had multifocal disease.

**Table 5:** Association of BRAFV600E and TERT overexpression with disease aggressiveness in 40 patients with thyroid cancer

Pathology	BRAFV600E	***P***-value	TERT	***P***-value
	***N*** = 20		***N*** = 10	
**Tumor type**	2	0.69	4	< 0.001
*ATC* vs *WDTC*				
**Stage**	8	0.626	7	0.025
*T3/4* vs *T1/2*	19	< 0.001	9	0.079
*N1* vs *N0*				
**LVI or PNI present**	10	0.047	3	0.702
**Extrathyroidal extension**	8	0.311	6	0.032
**Multifocal**	11	0.008	4	0.702
**Recurrence**	4	0.035	1	1

*ATC* anaplastic thyroid cancer; *LVI* lymphovascular invasion; *PNI* perineural invasion; *WDTC* well-differentiated thyroid cancer

## Discussion

Our study describes the utility of BRAFV600E and TERT ddPCR testing as a diagnostic and prognostic tool for thyroid FNAB. The current diagnostic standard of care for thyroid nodules meeting appropriate criteria is cytopathologic assessment of FNAB. A portion of these patients have indeterminate results, with the ATA reporting a risk of malignancy for Bethesda III of 5–15%, Bethesda IV of 15–30%, and Bethesda V of 60–75% (ATA guidelines). In such situations, the ATA has recommended molecular genetic testing may be performed to help further risk stratify. Of all genetics associated with well-differentiated thyroid cancer, BRAF and TERT mutations are the most robust prognosticators of aggressive disease [33]. We describe a simple, rapid, accurate and inexpensive tool for pre-operative molecular testing of BRAF and TERT.

This is the first study to assess the utility of combined BRAFV600E and TERT pre-operative testing from thyroid FNAB using ddPCR techniques. DdPCR is a nucleic acid detection technique that offers several advantages over other molecular tests. At an estimated cost for BRAFV600E ddPCR of $20.45 per FNAB, it is likely that ddPCR testing in conjunction with Bethesda grading may be more cost effective than commercially available molecular testing panels which can cost between $1675 (ThyGenX) and $4875 (Afirma GEC and MTC) per FNAB [27, 34]. DdPCR is able to provide rapid test results that are highly reproducible and accurate [22, 35]. Minimal nucleic acid is required, allowing assessment of smaller tissue samples than conventional molecular testing [22, 36].

Combining cytology with BRAFV600E and TERT testing increased the sensitivity of detecting a malignant diagnosis relative to cytology or molecular testing alone. Assessing ddPCR testing for BRAFV600E and TERT mutation as an adjunct to Bethesda IV-VI cytology increased the sensitivity from 72.5 to 80% while maintaining a stable specificity of 91.7%. Likewise, using BRAFV600E and TERT mutation testing as an adjunct to Bethesda V-VI cytology increased sensitivity from 70.0 to 80.0% while maintaining 100% specificity. In a 2019 study of 287 thyroid nodules with ARMS-qPCR, Zhao et al. also found that adding BRAFV600E assessment to standard cytology improved sensitivity with an increase from 75.7 to 92.3% (*P* < .001) [13]. However, a drop in specificity was appreciated from 89.2 to 84.6%. This was likely related to inherent error of FNAB or molecular techniques given that BRAFV600E assessment on FNAB cytology had lower specificity than on surgical pathology at 93.8 and 100%, respectively.

In keeping with current literature, BRAFV600E and/or high TERT levels were associated with aggressive or advanced stage pathology. BRAFV600E was significantly associated with nodal disease, lymphovascular or perineural invasion, multifocality and recurrence. Elevated TERT levels were significantly associated with arguably more disease including extrathyroidal extension and anaplastic thyroid cancer. In 2014, Liu and Xing were the first to investigate the utility of TERT mutations in thyroid nodule FNAB [15]. In an investigation of 308 thyroid nodules, eight nodules were positive for TERT, of which all demonstrated malignancy on surgical pathology. Nearly 80% of nodules with TERT mutations demonstrated aggressive behavior such as extrathyroidal invasion, metastases, or patient death. The combination of TERT and BRAFV600E had a sensitivity of 38.0% and specificity of 100% for thyroid cancer.

Qu et al. found that in PTC, multifocality is associated with more aggressive features and predicts a poorer prognosis [37]. In a study of 326 cases, Decaussin-Petrucci et al. also found not only were all nine TERT mutations associated with thyroid cancer, they were associated with aggressive features such as extrathyroidal extension and high stages [11]. A meta-analysis by Vuong et al. in 2017 found that BRAFV600E and TERT dual positivity was associated with increased rates of extrathyroidal extension, tumor recurrence, and mortality than isolated mutations [38]. The study supported the risk stratification of papillary thyroid carcinomas into three subgroups based on BRAF V600E and TERT promoter mutation positivity, with increasing aggressiveness from dual negativity, positivity in either mutation alone, to dual positivity [38]. Rengyun et al. found coexisting BRAFV600E and TERT promoter mutations had increased recurrence and mortality rates [14]. Xing et al. found that coexisting BRAF V600E and TERT mutations lead to worse clinicopathologic outcomes than isolated mutations with papillary thyroid cancer recurrence rates of 16.23% for BRAFV600E, 19.2% for TERT, and 68.6% for combined BRAFV600E and TERT [39]. Xing et al. proposed this synergistic effect may occur through BRAFV600E leading to an upregulation of TERT. TERT mutations increase transcription of the TERT promoter through upregulating E-twenty-six (ETS) complex transcription factors. BRAFV600E activates the mitogen-activated protein kinase pathway (MAPK) pathway, which is also believed to upregulate ETS complex transcription factors resulting in increased TERT expression. Song et al. corroborate these findings through transcriptomic analyses, confirming that TERT mRNA expression was increased as a result of upregulated ETS expression in the presence of BRAFV600E and TERT promoter mutation [40]. While research has focused on the relationship between mutations and malignancy, further study is needed to determine how various combinations of mutations affect tumor aggression and clinicopathology.

As suggested by Krasner et al., molecular testing is useful in predicting aggressive tumour variants and therefore may assist in planning the timing and extent of surgery [41]. For example, subtotal thyroidectomy may be best reserved for tumors which test negative for particularly aggressive genetic variants. Likewise, it may prove beneficial to incorporate mutational analysis into head and neck treatment protocols to guide management similar to that of p16+ oropharyngeal cancer. Further study is needed to understand how genetic testing may be best utilized to guide treatment decisions.

Our study is not without limitations. Our population was limited to a single tertiary centre, which may have created an inherent referral bias towards patients with more aggressive pathology. Furthermore, diagnostic yield of FNAB cytology and molecular testing are known to differ between sites [42]*.* The study cohort included a predominance of Bethesda II and VI patients, with relatively fewer Bethesda III - V patients (Table 1), limiting statistical analysis of Bethesda subgroups. Likewise, the study included a small number of dual positive BRAFV600E and TERT overexpressing tumors (Table 2), limiting analysis for this subset of patients. A multi-center study could further demonstrate the utility of ddPCR mutational testing for thyroid nodules while minimizing the effect of institutional differences and increasing the power to perform further subset analyses. Cytology and ddPCR from preoperative FNAB were compared to final surgical pathology. Evaluation of final surgical specimen with ddPCR would provide further insight into the utility of FNAB. This is especially true in genetically heterogenous tumors, where location of FNAB directly affects results [43, 44]. A limitation of evaluating TERT expression by ddPCR is that lymphocytes are also known to express TERT, and therefore lymphocyte contamination of the tumor sample could theoretically produce a false positive result [45]. This is of particular concern in thyroid nodules with concurrent thyroiditis. Given this concern, in situ hybridization techniques have been investigated showing promise in localizing TERT expression to specific cell types [46]. The risk of such a false positive affecting our sample is limited given that no nodules expressing TERT were benign on final pathology. While our sample included four tumors with thyroiditis (Supplementary Table 1), there does not appear to be a significant influence on TERT levels from lymphocytes, using a ddPCR cutoff threshold of 10%. While surgery is the recommended treatment for most thyroid nodules concerning for malignancy, the study sample did not capture those who did not undergo surgery leading to a potential sample bias. Also, while the study was conducted over 42 months, patients may go on to develop malignancy after the study window ended, which could affect the reported diagnostic accuracy.

## Conclusions

Combining cytology with ddPCR analysis of BRAFV600E and TERT can improve the diagnostic accuracy of thyroid FNABs. BRAFV600E and TERT overexpression demonstrated more aggressive clinicopathologic disease.

## Supplementary Information

**Additional file 1: Supplementary Table 1.** Histopathology and TERT ddPCR expression in benign thyroid nodules.

## Data Availability

The datasets used and/or analysed during the current study are available from the corresponding author on reasonable request.

## Abbreviations

ATA: American Thyroid Association
ATC: Anaplastic thyroid cancer
BRAFV600E: B-type raf proto-oncogene V600E
cDNA: complimentary deoxyribonucleic acid
ddPCR: Droplet digital polymerase chain reaction
dUTP: 2′-deoxyuridine, 5′ triphosphate
DTT: Dithiothreitol
EEF2: Eukaryotic elongation factor 2
ETS: E-twenty-six
FAM: 6-carboxyfluorescein
FNAB: Fine needle aspirate biopsy
FTC: Follicular thyroid cancer
HEX: Hexachloro-fluorescein
MAPK: Mitogen-activated protein kinase pathway
NLR: Negative likelihood ratios
NPV: Negative predictive value
PLR: Positive likelihood ratios
PPV: Positive predictive value
PTC: Papillary thyroid cancer
RNA: Ribonucleic acid
TERT: Telomerase reverse transcriptase
