# Supplementary material for: Utility of droplet digital polymerase chain reaction for TERT and BRAF mutational profiling of thyroid nodules
Source: BMC Cancer. 2021 Oct 26;21:1142. doi: 10.1186/s12885-021-08810-8 (PMC8547072; doi:10.1186/s12885-021-08810-8)
Supplement: Supplementary file 1 — Additional file 1: Supplementary Table 1. Histopathology and TERT ddPCR expression in benign thyroid nodules. [file 12885_2021_8810_MOESM1_ESM.docx]

**Supplementary Table 1.** Histopathology and TERT ddPCR expression in benign thyroid nodules

| **Histopathology** | **N** | **TERT LEVELS***   1. **<10% >10%** | | |
| --- | --- | --- | --- | --- |
| **Benign**  **Adenomatoid/hyperplasia**  **Follicular adenoma**  **Multinodular goitre**  **Thyroiditis** | 49  12  19  4 | 47  11  17  3 | 2  1  2  1 | 0  0  0  0 |

ATC, anaplastic thyroid cancer; FTC, follicular thyroid carcinoma; PTC, papillary thyroid

cancer. *0 value indicates undetectable levels by ddPCR.
